# Does Resilience Mediate the Association of Adverse Early Childhood Experiences With Emotional and Behavioural Problems?

**DOI:** 10.3389/ijph.2021.1604006

**Published:** 2021-11-25

**Authors:** Miriama Lackova Rebicova, Zuzana Dankulincova Veselska, Daniela Husarova, Andrea Madarasova Geckova, Danielle E. M. C. Jansen, Jitse P. van Dijk, Sijmen A. Reijneveld

**Affiliations:** ^1^ Department of Health Psychology and Research Methodology, Faculty of Medicine, PJ Safarik University, Kosice, Slovakia; ^2^ Graduate School Kosice Institute for Society and Health, PJ Safarik University, Kosice, Slovakia; ^3^ Department of Community and Occupational Health, University Medical Center Groningen, University of Groningen, Groningen, Netherlands; ^4^ Olomouc University Social Health Institute, Palacky University in Olomouc, Olomouc, Czech

**Keywords:** resilience, adverse childhood experiences, emotional problems, behavioral problems, adolescents

## Abstract

**Objectives:** To explore the role of resilience as a mediator in the association between adverse childhood experiences (ACE) and emotional and behavioural problems (EBP) among adolescents.

**Methods:** We used data from the Slovak 2018 Health Behaviour in School-aged Children study, comprising 2,839 adolescents aged 13–15 (mean age 13.93; 49.6% boys). We used multivariate linear regression performed on 5000 bootstrap samples adjusted for age, gender, family affluence to explore mediation of the associations between ACE (measured using the adapted Adverse Childhood Experience Questionnaire) and EBP (measured using the Strengths and Difficulties Questionnaire) by resilience (measured with the Child and Youth Resilience Measure).

**Results:** We found ACE [B = 0.78; 95% confidence interval (CI): 0.67|0.90] and resilience (B = −0.73; 95% CI: −0.79|−0.67) to be significantly associated with EBP. The association of ACE and EBP was mediated by resilience. The mediated indirect effect of resilience was ab = 0.25; 95% CI: 0.18|0.32.

**Conclusion:** Resilience seems to play a mediator role in the relationship between ACE and EBP. Helping adolescents with ACE to build and use internal and external sources of resilience can decrease the negative impact of ACE on EBP.

## Introduction

Adverse childhood experiences (ACE) regard a wide range of negative events that occur at a young age, such as abuse and/or neglect towards a child, domestic violence towards a youth’s mother, household substance abuse, household mental illness, parental separation/divorce, and other events [1–3]. ACE can cause serious emotional and behavioural problems (EBP) during both childhood and adult life [1, 2, 4–8]. Research also showed that cumulation of ACE among adolescents leads to serious juvenile delinquency and increased the incidence of crime in adolescence [9–11] and that specific ACE have been linked to delinquency and joining a gang related to the mental disorders that adolescents had [10, 12].

One of the factors that plays an important role in the case of ACE is resilience, which can be seen as a dynamic process supporting positive adaptation to negative events [13–19]. Overcoming severe ACE based on resources at the individual level is described in the theory of resilience. Resilience can be defined as the capacity of an individual to adapt to challenges that threaten the function and development of the individual. This capacity of an individual depends on his or her connections to other people and systems external to the individual [20, 21]. Resilience has been found to be a protective factor in relation to the development of EBP [22–28] and supports child flourishing while facing negative events [29]. This suggests a potentially mitigating role of resilience [30]. Previous literature has focused on resilience as a moderator in the association of ACE with EBP [31–33]; however, based on the existing knowledge, mediation might also be expected, but this has rarely been studied [31, 34]. Regarding mediation, recent findings show that those exposed to ACE have on average lower resilience [13]. Further, lower resilience was found to lead to more EBP among younger adolescents [23]. These findings can be interpreted as that previous experiences and rearing can be both a risk factor and a protective factor for EBP, similar to what has been shown for criminal behaviours of adolescents [9–11]. Finally, in one of the few previously published studies on resilience as a mediator, resilience was shown to mediate the relationship between family functioning and depression among adolescents from single parent families [34]. These findings support our hypothesis of resilience possibly having a role as mediator in the relations of ACE and EBP. Based on recent studies, resilience thus may be considered a mediator, but the evidence on this role of resilience is lacking.

The aim of our study was therefore to explore the role of resilience as mediator in the association between ACE and EBP among adolescents.

## Methods

### Sample and Procedure

We used data on resilience from 2,839 adolescents (mean age 13.93; 49.6% boys) from the Health Behaviour in School-aged Children (HBSC) study conducted in 2018 in Slovakia. These adolescents constituted a random sample of about 43.0% of all children participating in the HBSC study. We used three-step sampling to obtain a representative sample. In the first step, 140 larger and smaller elementary schools located in rural as well as in urban areas from all regions of Slovakia were asked to participate. These were randomly selected from a list of all eligible schools in Slovakia obtained from the Slovak Institute of Information and Prognosis for Education. The school response rate (RR) was 77.9%. Second, a random selection was taken from the resulting schools, leading to a representative sample of 8,405 adolescents, aged 11–15 years (mean age 13.43; 50.9% boys), from the fifth to ninth grades of the elementary schools; one class per grade was selected. Third, we obtained data on resilience from a random sample of the 13- to 15-year-olds among these adolescents, yielding 2,839 adolescents.

The study was approved by the Ethics Committee of the Medical Faculty at P. J. Safarik University in Kosice (16N/2017). Parents were informed about the study via the school administration and could opt out if they disagreed with their child’s participation. Participation in the study was fully voluntary and anonymous, with no explicit incentives provided for participation.

### Measures

Emotional and behavioural problems (EBP) were measured with the Strengths and Difficulties Questionnaire [35], from which we used the 20 difficulty items. The response categories were: not true (0), somewhat true [1], and certainly true [2], leading to scores ranging from 0 to 40, with a higher score indicating more problems. The sum score for overall difficulties can range from 0 to 40 [36]. Cronbach’s alpha for whole scale was 0.73 in our sample.

Adverse childhood experiences (ACE) were measured with Adverse Childhood Experience Questionnaire, which includes 11 questions on events: “Have you ever experienced any of the following serious events?” (Death of a brother/sister, Death of your father/mother, Death of somebody else you love, Long or serious illness of yourself, Long or serious illness of one of your parents or of someone else close to you, Problems of one of your parents with alcohol or drugs, Repeated serious conflicts or physical fights between your parents, Separation/divorce of your parents, Separation of your parents due work abroad, Moving to another house/flat, or city/village, Transfer to another school) [2, 37]. The response categories were “Yes” and “No”. We counted the number of ACE experienced and used this as a continuous score.

Resilience was measured using the Child and Youth Resilience Measure (CYRM-12) Child Version, which includes 12 items [38, 39] for adolescents 13 years and older (7th, 8th and 9th grade). The CYRM-12 is a measure of the resources (individual, relational, community and cultural) available to individuals that may bolster their resilience. The response categories ranged on a 3-point scale (No/Sometimes/Yes), leading to scores ranging from 12 to 36, with a higher score indicating higher resilience. Cronbach’s alpha in our sample was 0.84.

The Family Affluence Scale III (FAS III) was used as a measure of socioeconomic status. It consists of six questions: “Does your family own a car, van or truck?” (Yes/Yes, one/Yes, two or more); “Do you have your own bedroom for yourself?” (Yes/No); “How many computers does your family own?” (None/One/Two/More than two); “How many bathrooms (room with a bath/shower or both) are in your home?” (None/One/Two/More than two); “Does your family have a dishwasher at home?” (Yes/No); “How many times did you and your family travel out of your country for a holiday/vacation last year?” (Not at all/Once/Twice/More than twice). We converted the FAS summary scores to a final score, with a normal distribution and a range from 0 to 1. We then created tertile categories of low (0–0.333), medium (0.334–0.666) and high (0.667–1) socioeconomic position [40].

### Statistical Analyses

First, we described the sample using descriptive statistics. Second, we assessed the associations of ACE with EBP and of resilience with EBP (Model 1). Third, we repeated this assessment with adjustments for gender, age and family affluence (Model 2). Fourth, we explored the change in the associations of ACE with EBP after adding resilience (Model 3) in order to explore the mediation by resilience. For the last three steps we used multivariate linear models performed on 5000 bootstrap samples adjusted for age, gender and family affluence. Finally, we performed mediation analysis using PROCESS macro model 4 [41] and controlled for age, gender and family affluence on 5000 bootstrap samples. The indirect effect was calculated using the a*b product method, and bootstrapped 95% confidence intervals (CI) were calculated. Statistical analyses were performed using SPSS v.20.

## Results

The background characteristics of the sample are presented in Table 1. Our study sample consisted of 2,839 adolescents aged 13–15 years old [boys: 49.6% (1408)].

**TABLE 1:** Descriptive statistics of the sample (Health Behaviour in School-Aged Children - study, Slovakia 2018, 13–15 years old, *n* = 2839).

	Total*n* = 2839
Gender (N, %)	
Boys	1408 (49.6)
Age (N, %)	
13 years	957 (33.7)
14 years	1122 (39.5)
15 years	760 (26.8)
FAS (N, %)	
Low	828 (29.2)
Middle	858 (30.2)
High	1153 (40.6)
ACE (mean, SD) - range 0–11	2.42 (1.82)
Resilience (mean, SD) - range 12–36	29.25 (3.67)
Emotional and behavioural problems (mean, SD) - range 1–35	12.89 (5.53)

HBSC-study- Health Behaviour in School-Aged Children study; N- number of respondents; FAS- Family affluence; ACE- adverse childhood experiences; SD- standard deviation.

### Associations of Adverse Childhood Experiences and Resilience With Emotional and Behavioural Problems

We assessed the crude associations of the number of ACE and resilience scores with the number of EBP (Table 2, Model 1). We found more ACE to increase the number of EBP (B = 0.78; 95% CI: 0.67|0.90) and more resilience to decrease it (−0.73; −0.79|−0.67). B represents unstandardized coefficients from linear regression models on 5000 bootstrap samples.

**TABLE 2:** The mediation role of resilience in the association of numbers of adverse childhood experiences and emotional and behavioural problems; results from multivariate linear regression models leading to regression coefficients (B) and 95% confidence intervals (CI) performed on 5000 bootstrapped samples crude and adjusted for potential confounders (age, gender and family affluence) (Health Behaviour in School-Aged Children - study, Slovakia 2018, 13–15 years old, *n* = 2839).

	Model 1	Model 2	Model 3
	B (95% CI)	B (95% CI)	B (95% CI)
ACE	0.78 (0.67|0.90)***	0.73 (0.62|0.83)***	0.47 (0.38|0.57)***
Resilience	−0.73 (−0.79|−0.67)***		−0.69 (−0.74|-0.64)***

*p < 0.05.

**p < 0.01.

***p < 0.001.

Model 1 crude model–effect of ACE and of resilience.

Model 2 adjusted model–effect of ACE after adjustment for gender, age and family affluence.

Model 3 adjusted model–effect of ACE after adjustment for gender, age and family affluence controlled by resilience.

### Associations of Adverse Childhood Experiences With Emotional and Behavioural Problems Adjusted for Gender, Age and Family Affluence

In the next step we assessed the associations of the numbers of ACE and EBP with adjustment for potential confounders (Model 2). Again, we found each additional ACE to increase the numbers of EBP (0.73; 0.62|0.83), also after the adjustment.

### Mediation of the Associations Between Adverse Childhood Experiences and Emotional and Behavioural Problems by Resilience

**FIGURE 1:**
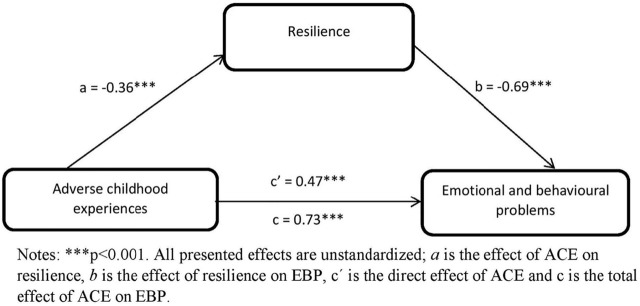
The mediation effect of resilience in the association of adverse childhood experiences and emotional and behavioural problems adjusted for age, gender and family affluence (Health Behaviour in School-Aged Children - study, Slovakia 2018, 13–15 years old, *n* = 2839).

We then assessed the role of resilience as a mediator in the association of numbers of ACE and EBP with adjustment for potential confounders (Model 3). We found that adding resilience to the model (−0.69; −0.74|−0.64) weakens the association of ACE with EBP (to 0.47; 0.37|0.57), suggesting a mediation role of resilience. Next, we assessed the strength of the mediation role of resilience in the association between numbers of ACE and EBP using the a*b product method, with adjustment for potential confounders (Figure 1). The results suggested that resilience mediated the association of ACE with EBP with an indirect effect of ab = 0.25 (0.18|0.32).

## Discussion

The aim of our study was to explore the role of resilience as a mediator in relation to the association between ACE and EBP among adolescents. Our results show that resilience decreased the probability of EBP among adolescents, and resilience mediates the association between ACE and EBP among adolescents. Our study is one of the first to analyse the mediating role of resilience on the relationship between ACE and EBP among adolescents.

First, we found that ACE increased and resilience decreased the probability of EBP among adolescents, confirming previous findings for both ACE [42] and for resilience [23, 43]. These findings can be interpreted as that previous experiences and rearing can be both a risk factor and a protective factor for EBP, similar to what has been shown for criminal behaviours of adolescents [9–11]. Positive adaptation to negative events may thus reduce risks of EBP. This suggests that ACE and lower resilience may be among the reasons for an increased occurrence EBP among adolescents.

Next, our findings support the role of resilience as a mediator in the relation between ACE and EBP, confirming previous findings suggesting such mediation [13, 14, 23], but now in an analysis covering the full chain of mediation. An explanation may be that adolescents with ACE do not have internal resources (personality) and external sources (family, peer, school, neighbours, community) of resilience due to ACE and the emotional distress associated with ACE. ACE overwhelmed them so much that they are unable to accept help from family, friends or school ([44, 45]), i.e., they cannot adapt positively. The presence of ACE and the lack of internal and external sources of resilience result in an increased occurrence of EBP in adolescents. If protective factors are not present in adolescents, this may lead to an increased occurrence of EBP, which leads to an early initiation into delinquent behavior [10, 12]. Our findings are consistent with research that points out the importance of building a child’s resilience and family protective factors to both attenuate the impact of ACE [29, 30]. Support for the building of internal and providing external resources of resilience seems to have a crucial role in the healthy emotional and behavioural development of adolescents exposed to ACE.

### Strengths and Limitations

The main strengths of our study are its large nationally representative sample and its use of the well-established HBSC methodology. This study also has some limitations. Our research had a cross-sectional design, which hinders conclusive inferences about causality. Furthermore, we used self-reported data from adolescents, which may lead to e.g., the underreporting of ACE. We used well-validated questionnaires for this, but we cannot exclude some underestimation of their occurrence. The third limitation of this study is that we used the ACEQ, which does not include questions about childhood abuse. This topic is evidently hard to measure based on questionnaires but could have even stronger effects than the ACE that we included. This means that we may have underestimated the real associations. The fourth limitation is that we did not assess whether the association and mediation as found is stronger for high levels of either the outcome (dependent) or of the determinant (independent). However, if limiting the range of determinant or outcome, then typically the association will weaken.

### Implications

We found that resilience plays a mediating role among adolescents with ACE in the incidence of EBP. Our study suggests that it is important to help adolescents with ACE build and be able to use internal and external sources of resilience, as we know these sources can decrease the negative impact of ACE on EBP. Resilience may affect the association of ACE and EBP differently depending on the specific type of ACE. Future research could focus on specific ACE in relation to EBP; and on the role of resilience in relation to specific ACE and EBP. Our results imply that better communication between family and school, e.g., classroom teachers and school psychologists, could support external sources of resilience in adolescents with EBP. Future research could take a closer look at building resilience at a younger age, which may then prevent EBP in adolescents. A longitudinal study may be useful for examining the causality of these findings. Longitudinal research could shed more light on this topic for better understanding the building of resilience already in childhood.

### Conclusion

Resilience plays an important role in the relationship between ACE and EBP. We found that resilience is a mediator in the association between ACE and EBP among adolescents.
